# Caloric restriction reduces proteinuria in male rats with established nephropathy

**DOI:** 10.14814/phy2.15942

**Published:** 2024-03-05

**Authors:** J. W. A. Sijbesma, A. van Waarde, A. Klooster, I. Kion, R. H. J. A. Slart, A. A. Lammertsma, B. Lima Giacobbo, H. H. Boersma, R. A. J. O. Dierckx, H. van Goor, S. J. L. Bakker

**Affiliations:** ^1^ Department of Nuclear Medicine and Molecular Imaging University Medical Center Groningen, University of Groningen Groningen The Netherlands; ^2^ Department of Pathology Pathologie Friesland Leeuwarden The Netherlands; ^3^ Department of Biomedical Photonic Imaging, Faculty of Science and Technology University of Twente Enschede The Netherlands; ^4^ Department of Clinical Pharmacy and Pharmacology University Medical Center Groningen, University of Groningen Groningen The Netherlands; ^5^ Department of Pathology and Medical Biology University Medical Center Groningen, University of Groningen Groningen The Netherlands; ^6^ Department of Nephrology University Medical Center Groningen, University of Groningen Groningen The Netherlands

**Keywords:** ^13^[N]ammonia PET, caloric restriction, protein excretion, proteinuria

## Abstract

Reducing proteinuria is a crucial approach in preventing kidney function loss. Previous preclinical studies indicated that caloric restriction (CR) imposed at a young age protects against age‐related proteinuria. However, these studies have not explored CR in established renal disease. Therefore, this study aimed to investigate the impact of CR on established proteinuria. Rats, aged 12 ± 2 weeks, were administered 2.1 mg/kg of Adriamycin. Six weeks after injection, protein excretion was measured, and a [^13^N]ammonia positron emission tomography (PET) scan was conducted to assess kidney perfusion. After 7 weeks rats were divided into four groups: ad libitum (AL) and CR groups fed either a 12% or a 20% protein diet. All groups were treated for 12 weeks. Blood pressure was measured and a second PET scan was acquired at the end of the study. The animals subjected to CR exhibited a 20.3% decrease in protein excretion (*p* = 0.003) compared to those in the AL groups. Additionally, blood pressure in the CR group was 21.2% lower (*p* < 0.001) than in the AL groups. While kidney function declined over time in all groups, the 20% CR group demonstrated the smallest decline. Thus CR effectively reduces urinary protein excretion and lowers blood pressure in rats with established proteinuria.

## Graphical abstract

This study shows that caloric restriction reduces proteinuria and blood pressure in rats with established renal dysfunction even in groups which were corrected for protein and sodium loss due to caloric restriction.The findings prove that caloric restriction is a potential treatment for proteinuria in rats with established renal disease.

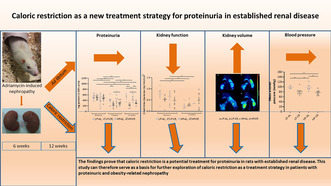

## 1 INTRODUCTION

Proteinuria plays a crucial role as a diagnostic parameter for predicting the progression of chronic kidney disease (CKD). In addition to its role as a predictor, proteinuria can also contribute to kidney damage and the loss of kidney function. Consequently, reducing proteinuria is a significant treatment strategy to prevent or slow down the decline in kidney function and to minimize renal damage (Ruggenenti et al., 2003).

One approach to treat proteinuria is by adopting a low protein diet, which effectively lowers protein intake. This reduction in dietary protein intake helps decrease intraglomerular pressure (Kalantar‐Zadeh et al., 2020), reduces hypertrophy, and minimizes protein leakage into the urine, particularly in non‐diabetic CKD patients (Kaysen & Odabaei, 2013; Wang et al., 2017). However, it's important to note that the effectiveness of dietary protein restriction is still a subject of debate (Ikizler, 2009). Very low protein diets, in particular, may adversely affect the nutritional status of patients (Ikizler, 2009) and are associated with an increased risk of mortality (Menon et al., 2009).

Besides a low‐protein diet, other dietary interventions have demonstrated the ability to delay the progression of kidney disease. Multiple studies have revealed that a ketogenic diet, characterized by low carbohydrate intake, can reverse nephropathy (Poplawski et al., 2011) and significantly reduce proteinuria (Lin, 2009). Nonetheless, ketogenic diets carry an increased risk of hypercholesterolemia (Best et al., 2000; Dhamija et al., 2013) and cardiomyopathy (Best et al., 2000).

Another promising treatment strategy is caloric restriction (CR). CR is known for lowering body weight due to reduced caloric intake, and for lowering steady‐state levels of oxidative stress and associated decline in function (Saad, 2020; Sohal & Weindruch, 1996; Weindruch & Sohal, 1997), especially in organs with high oxidative demand like kidneys (Chen et al., 2008). CR is linked to a reduction in age‐associated decline in renal function (Davis et al., 1983; Keenan et al., 2000; Wiggins et al., 2005).

Preclinical studies have indicated that CR, when initiated at a young age, offers protection against age‐related proteinuria (Keenan et al., 2000; Wiggins et al., 2005). However, these studies have primarily focused on the preventive aspects of CR against age‐related changes induced by oxidative stress, rather than evaluating the effects of CR as a treatment in established renal disease (Chen et al., 2008; Davis et al., 1983; Gumprecht et al., 1993; Keenan et al., 2000; McKiernan et al., 2007; Wiggins et al., 2005; Xu et al., 2015). Considering that kidney disease is often diagnosed at a later stage, successful treatments for established renal disease are more relevant.

Other studies have not accounted for the reduced protein intake resulting from CR (Klooster, 2013), making it challenging to isolate the effects of CR from protein restriction. Therefore, the objective of this study was to assess whether CR treatment in rats with established Adriamycin‐induced nephropathy could effectively reduce proteinuria and mitigate the decline in kidney function.

To distinguish the effects of CR from protein restriction, we fed the animals either a low‐protein or a high‐protein diet. If successful, these findings could provide a basis for further investigation of the potential use of CR as an additional treatment strategy for patients with renal disease.

## 2 MATERIALS AND METHODS

### 2.1 Animals

Male Wistar rats (strain HsdCpb:WU) (*n* = 56; aged 12 ± 2 weeks) were obtained from Envigo (Venray, The Netherlands) and acclimatized for a period of 7 ± 2 days. The rats were housed at a temperature of 21 ± 2°C and maintained under a 12‐h light/12‐h dark regime. Prior to the start of the treatment, the animals were fed with Rodent Breeder (RB) 12% diet (824245, Special Diets Services, Witham, UK).

The experimental protocol was conducted in accordance with the ARRIVE guidelines and was approved by the Central Committee on Animal Experiments of The Netherlands (license number AVD105002016663) and the animal welfare body of the University Medical Center Groningen (protocol 16663‐01‐001), in compliance with directive 2010/63/EU of the European Parliament.

### 2.2 Experimental design

Anesthetized rats (using a mixture of oxygen and 2%–5% isoflurane) were intravenously injected with Adriamycin (2.1 mg/kg body weight, Doxorubicin, Accord, Middlesex, UK, administered over 10 ± 2 s) to induce nephropathy. Biochemical levels (plasma and urinary creatinine, sodium, and protein) were measured at 6 weeks after injection (baseline) to confirm the presence of renal disease. At Week 7 after injection, positron emission tomography (PET) scans using [^13^N]ammonia were performed on anesthetized animals to assess kidney perfusion. Following the scan, the animals were randomly assigned to four groups, that is two ad libitum groups fed with either a low protein diet ([diet code: 824245] 12% protein, LP‐AL) or a high protein diet ([824250] 20% protein, HP‐AL), and two CR groups fed with either a low protein diet ([824252] 12% protein LP‐CR) or a high protein diet ([824254] 20% protein, HP‐CR). All diets were obtained from Special Diets Services (SDS), Witham, UK.

CR treatment was administered by feeding the animals daily 60% (13.8 g) of their normal food intake (23.0 g) calculated based on a pilot study. To account for the reduced protein intake caused by CR and to distinguish the effect of CR from protein restriction, the HP‐AL and the HP‐CR groups were included. Therefore, the diet of the LP‐AL group contained 12% protein, the diet of the LP‐CR group contained 7.32% protein after CR, the diet of the HP‐AL group contained 20% protein, and the diet of the HP‐CR group contained 12% protein after CR. Additional vitamins and minerals were added to the diets of the CR groups to prevent deficiencies. The diets (detailed in Table S1) were isocaloric to facilitate comparison between groups.

All groups were fed with their respective diets for 12 weeks. Body weight was measured weekly, and biochemical parameters were assessed at Weeks 9 and 12. The [^13^N]ammonia PET scan was repeated at the end of the study, along with a blood pressure measurement. Two days after the last PET scan, animals were euthanized by perfusion under deep anesthesia and kidneys were collected for immunohistochemistry.

### 2.3 Biochemical measurements

At baseline and Weeks 9 and 12, the rats were placed in metabolic cages for 24‐h urine collection. After the collection period, a blood sample was taken. Plasma and urine creatinine, urinary protein concentration, and sodium levels were measured using the Roche Modular platform (Roche Diagnostics GmbH, Mannheim, Germany) following the routine procedures in our Institution.

Creatinine clearance was calculated by multiplying the urinary creatinine concentration by the 24‐h urine volume, dividing it by the plasma creatinine level, and then correcting it for kidney volume (in cm^3^) derived from the PET scans.

### 2.4 PET and blood pressure procedures

On the day of the scan, the animals were anesthetized using a mixture of oxygen (95%) and isoflurane (5% for induction and ≤2% for maintenance) and positioned in a dedicated small animal PET camera (Focus 220, Siemens Molecular Imaging/Concorde Microsystems Inc., Knoxville, TN, USA) with the kidneys within the field of view. To correct for attenuation and scatter, a transmission scan (515 s) was performed using a ^57^Co point source.

[^13^N]Ammonia is generated through the irradiation of water supplemented with ethanol with protons using the 16O (p, α) ^13^N nuclear reaction. Following irradiation, the liquid undergoes filtration through a QMA SEPPAK ion‐exchange cartridge to eliminate any residual [^13^N]nitrite and [^13^N]nitrate. The resulting [^13^N]ammonia solution is collected in a sterile vial containing a 0.9% NaCl solution. The diluted [^13^N]ammonia solution is then passed through two consecutive 0.22 μm sterilization filters before being transferred into a syringe for injection. The animals were then intravenously injected with [^13^N]ammonia (45.3 ± 5.6 MBq) over a period of 60 s using an infusion pump (Pump 11 Elite syringe pump, Harvard Apparatus, Holliston, MA, USA) and scanned for 10 min. Following the scan, each animal was returned to a heated home cage, and the treatment was initiated. After 12 weeks of treatment, the PET procedure was repeated, followed by a 5‐min blood pressure measurement using the femoral artery and a Cardiocap (Datex Instrumentarium Corp., Helsinki, Finland).

PET data were reconstructed using an OSEM2D (ordered subsets expectation maximization) reconstruction algorithm with Fourier rebinning, 4 iterations, and 16 subsets. The voxel size was 0.47 × 0.47 × 0.80 mm, and the spatial resolution within the field of view was 1.5 mm. The scans were corrected for decay, random coincidences, scatter, and attenuation. The reconstructed PET images were analyzed using PMOD version 4.1 (PMOD Technologies Ltd., Zürich, Switzerland). Regions of interest (ROI) were drawn around the cortical and medullar regions in five consecutive slices (Juárez‐Orozco et al., 2015). An additional ROI was drawn within the lumen of the abdominal part of the aorta to obtain an image‐derived input function. A one‐tissue compartment model, incorporating additional blood volume for the first two min of the total acquisition time, was used to determine kidney perfusion (mL/min/cm^3^). Furthermore, a ROI was drawn around both kidneys to determine kidney volume (cm^3^).

### 2.5 Immunohistochemistry

Deparaffinized kidney sections (3/4 μm) were stained for pro‐fibrosis with α‐smooth muscle actin (α‐SMA [Monoclonal Anti‐Actin, α‐Smooth Muscle, A2547, Sigma‐Aldrich, Burlington, MA, USA]), for periglomerular and glomerular macrophages with ED1+ (MCA341R, Bio‐Rad Laboratories, Veenendaal, The Netherlands), and for focal glomerulosclerosis with periodic acid‐Schiff (PAS [Periodic acid ≥99.5%, AnalaR NORMAPUR® analytical reagent, Sigma‐Aldrich, Burlington, MA, USA]). Stained sections were scored following procedures described previously (Melenhorst et al., 2006; Poosti et al., 2012; Snijder et al., 2014; van Goor et al., 1991).

### 2.6 Statistics

For repeated measurements (body weight, biochemical measurements, PET scans, and blood pressure), the generalized estimating equations (GEE) model was used to account for both repeated measurements in the design and missing data. The independent correlation matrix was selected for the analysis, and the Wald test was used to report *p*‐values, without correction for multiple comparisons. Parametric data for single time points (immunohistochemistry data) were tested using ANOVA and post hoc Tukey. A *p‐*value smaller than 0.05 was considered statistically significant.

## 3 RESULTS

### 3.1 Different growth curves between the CR and AL groups

Despite the low dose of Adriamycin only 24 animals out of the initial 56 animals, survived until the end of the study. Animals without renal dysfunction (*N* = 2) were excluded from the experiment. Due to ethical reasons, 30 animals were euthanized (overdose of pentobarbital, Euthasol®, AST farma B.V., Oudewater, The Netherlands) during the treatment period due to epistaxis, severe hematuria, and cachexia (as shown in Table S2).

**FIGURE 1:**
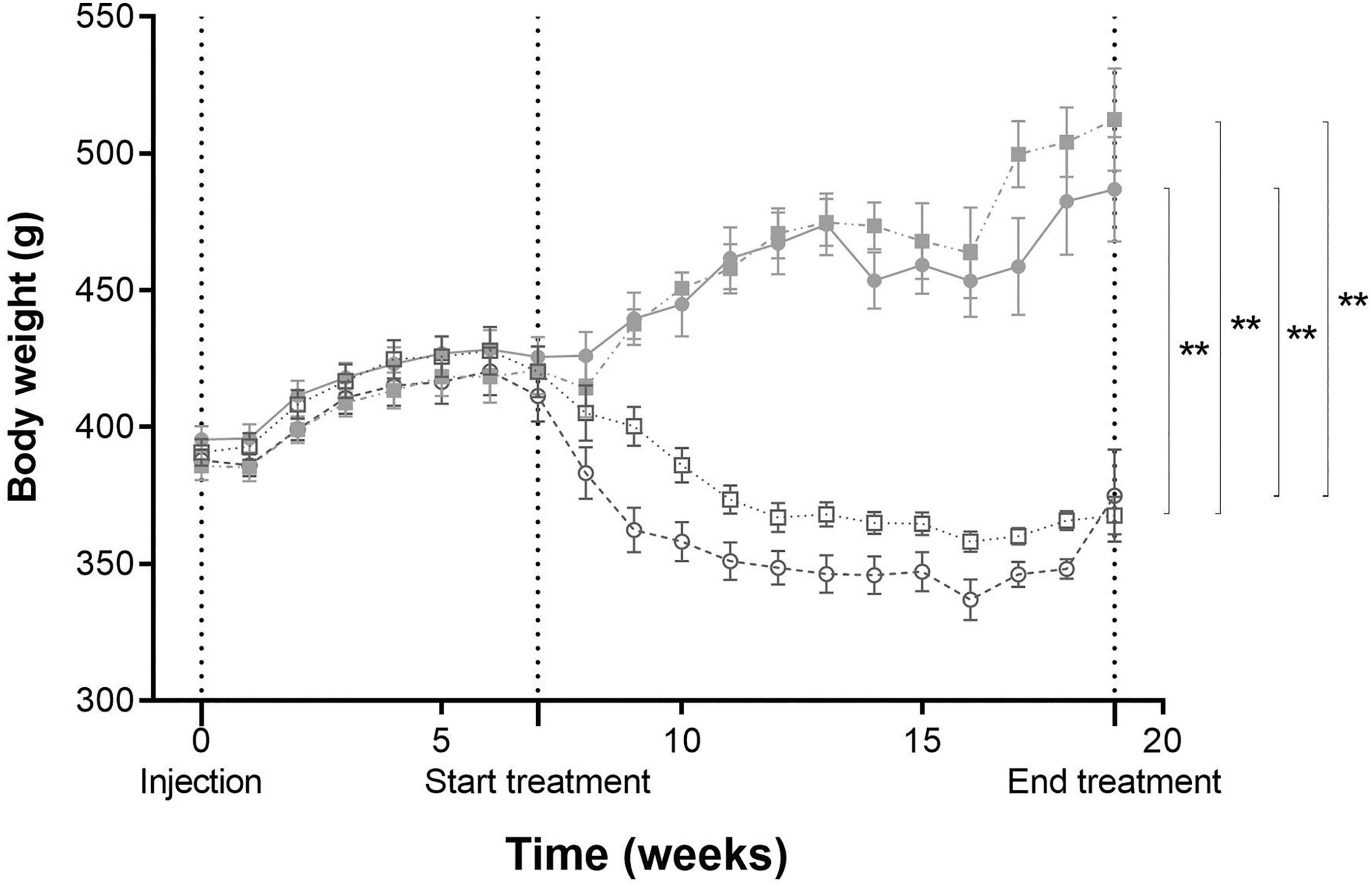
Weekly measurements of body weight in each group from the moment of the Adriamycin injection until the end of the study at week 19. ● LP‐AL, 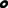 LP‐CR, ■ HP‐AL, 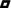 HP‐CR. Data are presented as mean ± SEM. * *p* < 0.05, ** *p* < 0.005.

The body weight of all groups was comparable prior to the administration of Adriamycin injection (390 ± 5 g) and before the initiation of treatment (418 ± 7 g) (as shown in Figure 1). By Week 10, the body weight of the rats treated with CR was significantly lower compared to the AL fed animals (*p* < 0.005). The body weight of the CR‐treated rats continued to decrease until Week 16 and then stabilized. Conversely, the AL‐fed animals showed a continued increase in body weight. At the end of the study, the body weight of the LP‐CR group was 33% lower (*p* < 0.005) compared to the LP‐AL group, and the HP‐CR group had a 25% lower body weight compared to the HP‐AL group (*p* < 0.005).

### 3.2 CR‐treated rats show lower levels of proteinuria

**FIGURE 2:**
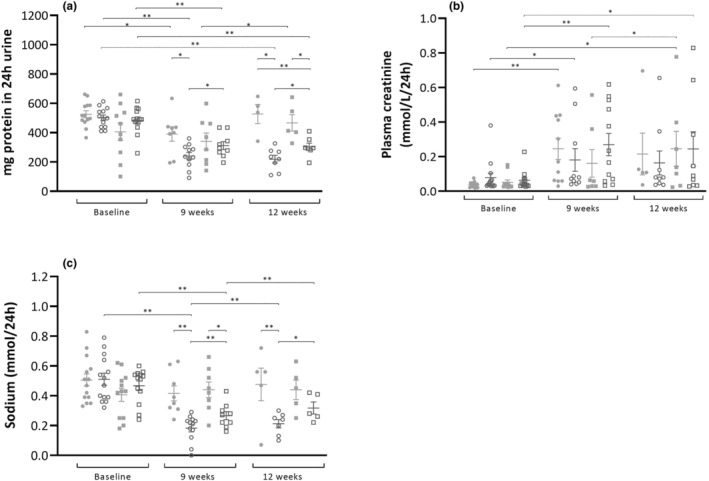
Biochemical measurements performed in 24‐h rat urine per time point per group, to assess kidney functionality. (a) Urinary protein excretion, (b) plasma creatinine (c) urine sodium excretion. ● LP‐AL, 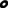 LP‐CR, ■ HP‐AL, 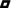 HP‐CR. Data are presented as mean ± SEM. * *p* < 0.05, ** *p* < 0.005.

The levels of proteinuria at the beginning of the treatment (as shown in Figure 2a) were similar in all groups (479 ± 17 mg/24 h). When considering the CR effect, proteinuria levels were significantly lower overall in animals treated with CR compared to those fed AL (442 ± 22 vs. 343 ± 13 mg, *p* < 0.005, Wald *χ* ^2^ = 15.4). Examining the diet effect overall, no significant differences in proteinuria were observed between animals treated with a LP or a HP diet (401 ± 17 vs. 385 ± 19 g, *p* = 0.53, Wald *χ* ^2^ = 0.39).

In addition to the treatment effect, a temporal effect was observed (*p* < 0.05). Proteinuria levels decreased from 479 ± 17 to 320 ± 120 mg at Week 9 and increased to 379 ± 21 mg at the end of the study. In a more detailed examination using GEE analysis (between groups per time point), significantly higher levels of proteinuria were observed in the LP‐AL group compared to the HP‐CR group at Week 12 (527 ± 57 vs. 303 ± 23 mg, *p* < 0.005). However, this effect did not reach statistical significance at Week 9 (391 ± 47 vs. 312 ± 26, *p* = 0.137).

Significantly lower levels of proteinuria were observed in the LP‐CR group at Week 9 (391 ± 47 vs. 240 ± 24 mg, *p* < 0.005) and at the end of the study (427 ± 101 vs. 196 ± 31 mg, *p* < 0.05) compared to the LP‐AL group. Between the HP‐AL and HP‐CR groups, a difference was noted only at the end of the study (466 ± 50 vs. 303 ± 23 mg, *p* < 0.05). Furthermore, significantly lower levels of proteinuria were measured in the LP‐CR group compared with the HP‐CR group at Week 9 (240 ± 25 vs. 312 ± 25 mg, *p* < 0.05) and at the end of the study (195 ± 31 vs. 303 ± 23 mg, *p* < 0.05).

No significant effect in plasma creatinine (shown in Figure 2b) was observed from the CR treatment (*p* = 0.944) or diet (*p* = 0.635), but a significant increase (*p* < 0.05) over time was noted in all groups. However, the increase was less prominent in the CR groups (compared to baseline, 5.8 and 4.7‐fold higher in the LP‐AL and HP‐AL groups vs. 3.0 and 3.8‐fold higher in the LP‐CR and HP‐CR groups).

Sodium excretion (shown in Figure 2d) in the AL groups did not change significantly between the start and end of the study. Sodium excretion in the CR groups, however, dropped over time, with a decrease of 62 ± 19% in the LP‐CR group (*p* < 0.005) and a decrease of 25 ± 44% in the HP‐CR group (*p* < 0.005).

### 3.3 Decrease in kidney function for all rats but less severe in HP‐CR‐treated rats

**FIGURE 3:**
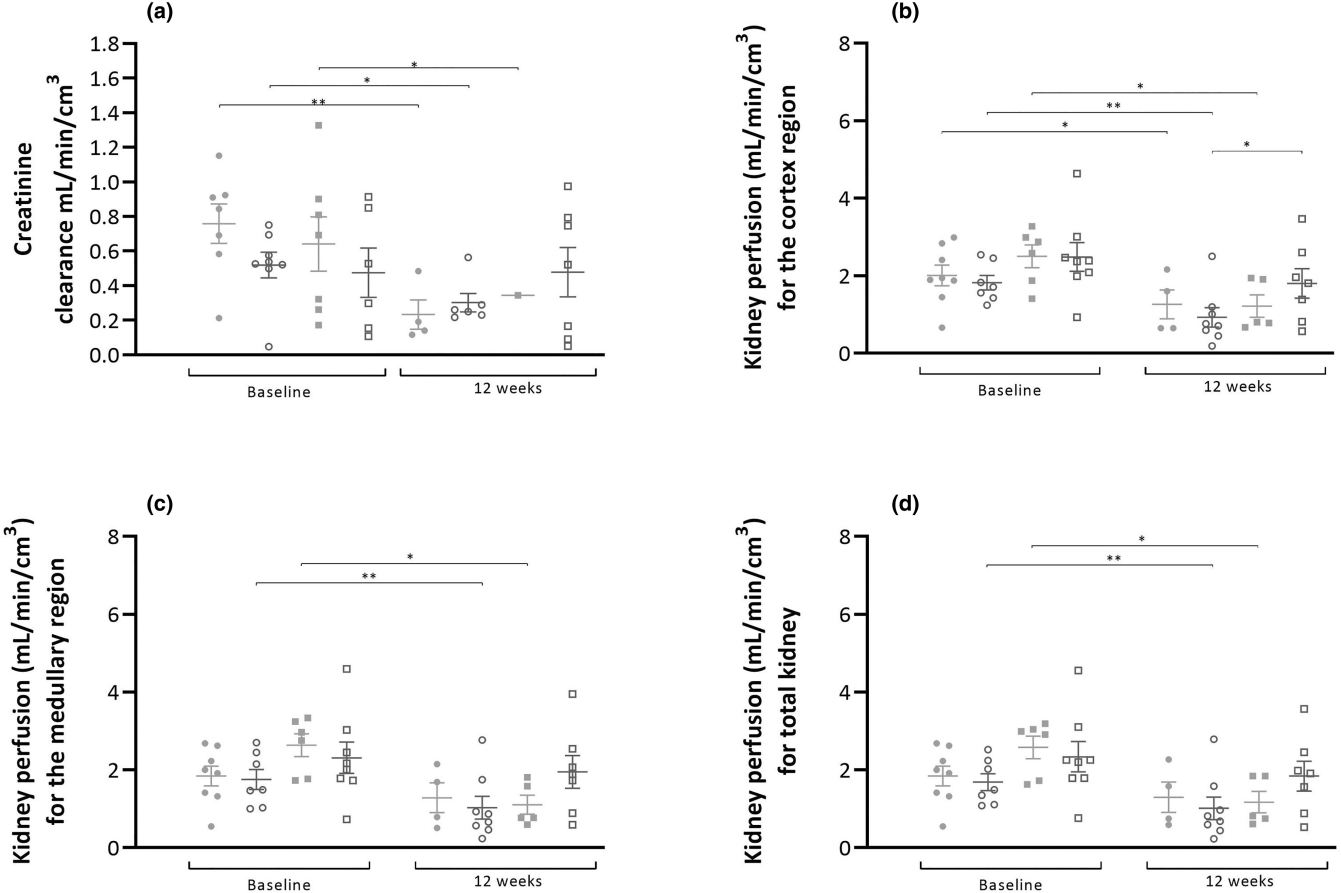
(a) Creatinine clearance at baseline and at the end of the study. Creatinine clearance is expressed as mL per minute per cm^3^ of kidney (obtained from the PET scans). Kidney perfusion in the cortex (b), medullary region (c) and total kidney (d) at baseline and at the end of the study. Perfusion is expressed as mL/min/cm^3^. ● LP‐AL, 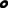 LP‐CR, ■ HP‐AL, 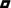 HP‐CR. Data are presented as mean ± SEM. * *p* < 0.05, ** *p* < 0.005.

Creatinine clearance (mL/min/cm^3^) (shown in Figure 3a) did not demonstrate a treatment effect (*p* = 0.504) or a diet effect (*p* = 0.683). However, a clear temporal effect was observed (*p* < 0.01), with 0.60 mL/min/cm^3^ at baseline and 0.34 mL/min/cm^3^ at the end of the study. When comparing creatinine clearance before and after treatment (Table 1), the smallest decline in kidney function was found in the HP‐CR group (−1.6 ± 16.4%, *p* = 0.99).

**TABLE 1:** Relative difference in creatinine clearance and kidney perfusion (mL/min/cm^3^) between baseline and end of the study. Data are reported as mean ± SD.

		LP‐AL	LP‐CR	HP‐AL	HP‐CR
Creatinine clearance (mL/min/cm^3^)					
% Δ		74 ± 19 *p* < 0.005	44 ± 28% *p* < 0.05	46 ± 22 *p* < 0.05	−1.6 ± 16.4 *p* = 0.99
Kidney perfusion (mL/min/cm^3^)					
Cortex	% Δ	−27 ± 34 *p* = 0.037	−46 ± 28 *p* < 0.001	−26 ± 54 *p* = 0.008	−21 ± 57 *p* = 0.172
Medullary region	% Δ	−10 ± 56 *p* = 0.156	−36 ± 35 *p* < 0.001	−39 ± 42 *p* = 0.007	−10 ± 69 *p* = 0.491
Total kidney	% Δ	−27 ± 34 *p* = 0.154	−46 ± 42 *p* = 0.006	−32 ± 49 *p* = 0.003	−17 ± 62 *p* = 0.324

Kidney perfusion (mL/min/cm^3^) (shown in Figure 3b–d) decreased over time in all groups, affecting both cortical and medullary region as well as the total kidney (Table 1). The decrease in kidney perfusion was highest in the LP‐CR and HP‐AL groups, while the LP‐AL and HP‐CR groups showed the lowest decrease. In the cortex, a significant difference was found between the LP‐CR and HP‐CR groups (*p* < 0.05). However, no differences were found between groups in the medullary region or the total kidney.

### 3.4 CR‐treated rats show smaller kidney volume and urine production

**FIGURE 4:**
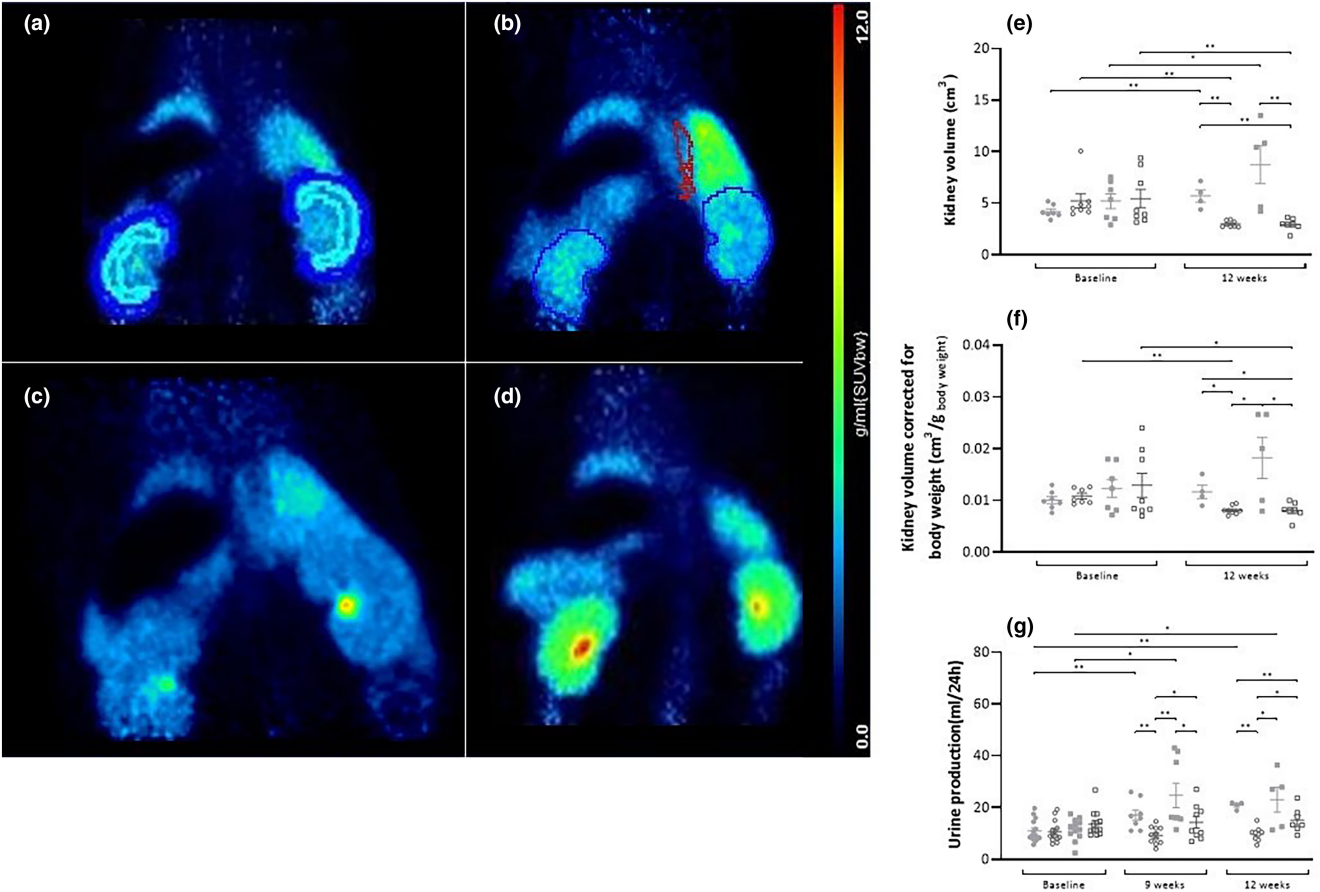
[^13^N]ammonia PET scans (expressed as standardized uptake value (SUV)) of rats with the kidneys in the field of view, made at the end of the study. (a) a rat from the LP‐AL group with a ROI drawn around the cortical and medullar region to determine kidney perfusion, (b) a rat from the LP‐CR group with a ROI drawn around the abdominal part of the aorta for an image‐derived input function and a ROI around both kidney to determine kidney volume, (c) a rat from the HP‐AL group with enlarged kidneys, (d) a rat from the HP‐CR group. (e) Total kidney volume per time point per group. (f) Total kidney volume corrected for body weight (cm^3^/g _body weight_) per time point per group. (g) Urine production in mL/24 h. ● LP‐AL, 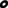 LP‐CR, ■ HP‐AL, 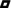 HP‐CR. Data are presented as mean ± SEM. * *p* < 0.05, ** *p* < 0.005.

The kidney volume, as determined by PET imaging (as shown in Figure 4a–d), significantly increased at the end of the study in both AL groups compared to the start of treatment: 49 ± 24% in the LP‐AL group and 99 ± 115% in the HP‐AL group. In contrast, a decrease in kidney volume was observed (Figure 4e) in both CR groups: 33 ± 11% in the LP‐CR group and 25 ± 23% in the HP‐CR group. No significant differences were found between the CR groups or between the AL groups.

When correcting kidney volume for body weight (cm^3^/g body weight) (Figure 4f), we no longer observed an increase in the AL groups, but a decrease persisted in the CR groups between baseline and the end of the study. Additionally, at the end of the study, CR groups had a significantly lower corrected kidney volume compared to the AL groups (*p* < 0.005).

Urine production (Figure 4g) was similar for all groups at baseline but increased over time from 10.9 ± 1.1 to 20.9 ± 0.6 mL/24 h (*p* < 0.005) in the LP‐AL group and from 11.6 ± 1.2 to 23.0 ± 4.3 mL/24 h (*p* < 0.05) in the HP‐AL group. At Weeks 9 (*p* < 0.05) and 12 (*p* = 0.005), a significant difference was observed between the two CR groups.

### 3.5 Reduced blood pressure for CR‐treated animals

**FIGURE 5:**
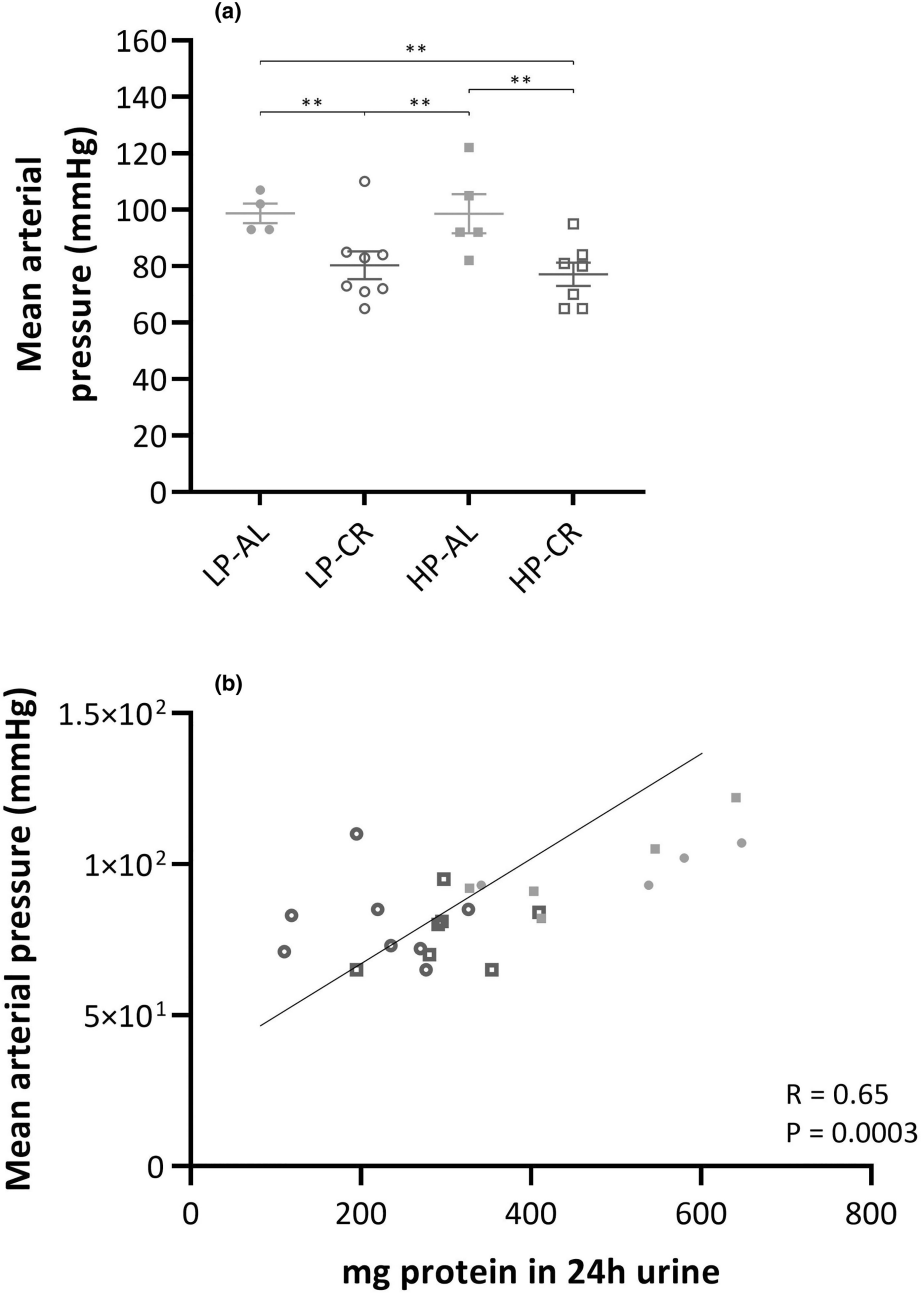
(a) Mean arterial pressure (mmHg) per group at the end of the study. (b) Correlation between mean arterial pressure and proteinuria. ● LP‐AL, 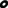 LP‐CR, ■ HP‐AL, 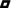 HP‐CR. Data are presented as mean ± SEM. * *p* < 0.05, ** *p* < 0.005.

The mean arterial blood pressure (Figure 5a) in animals treated with CR was 75.1 ± 3.3 mmHg, representing a 21.1% decrease (*p* < 0.005) compared to animals fed ad libitum, whose mean arterial blood pressure was 95.0 ± 2.8 mmHg. Upon closer examination between groups, we noted significantly lower values in LP‐CR (80.4 ± 4.6) compared to LP‐AL (98.8 ± 3.0, *p* < 0.005) and HP‐AL (98.6 ± 6.1, *p* < 0.05), as well as in HP‐CR (77.1 ± 3.9) compared to LP‐AL (*p* < 0.005) and HP‐AL (*p* < 0.005). No significant differences were found between the CR and AL groups.

A correlation (Figure 5b) was observed between blood pressure and proteinuria, with a correlation coefficient (*r*) of 0.65, indicating a moderate positive correlation between the two variables (*p* < 0.005).

### 3.6 Lower number of glomerular macrophages in CR‐treated animals

**FIGURE 6:**
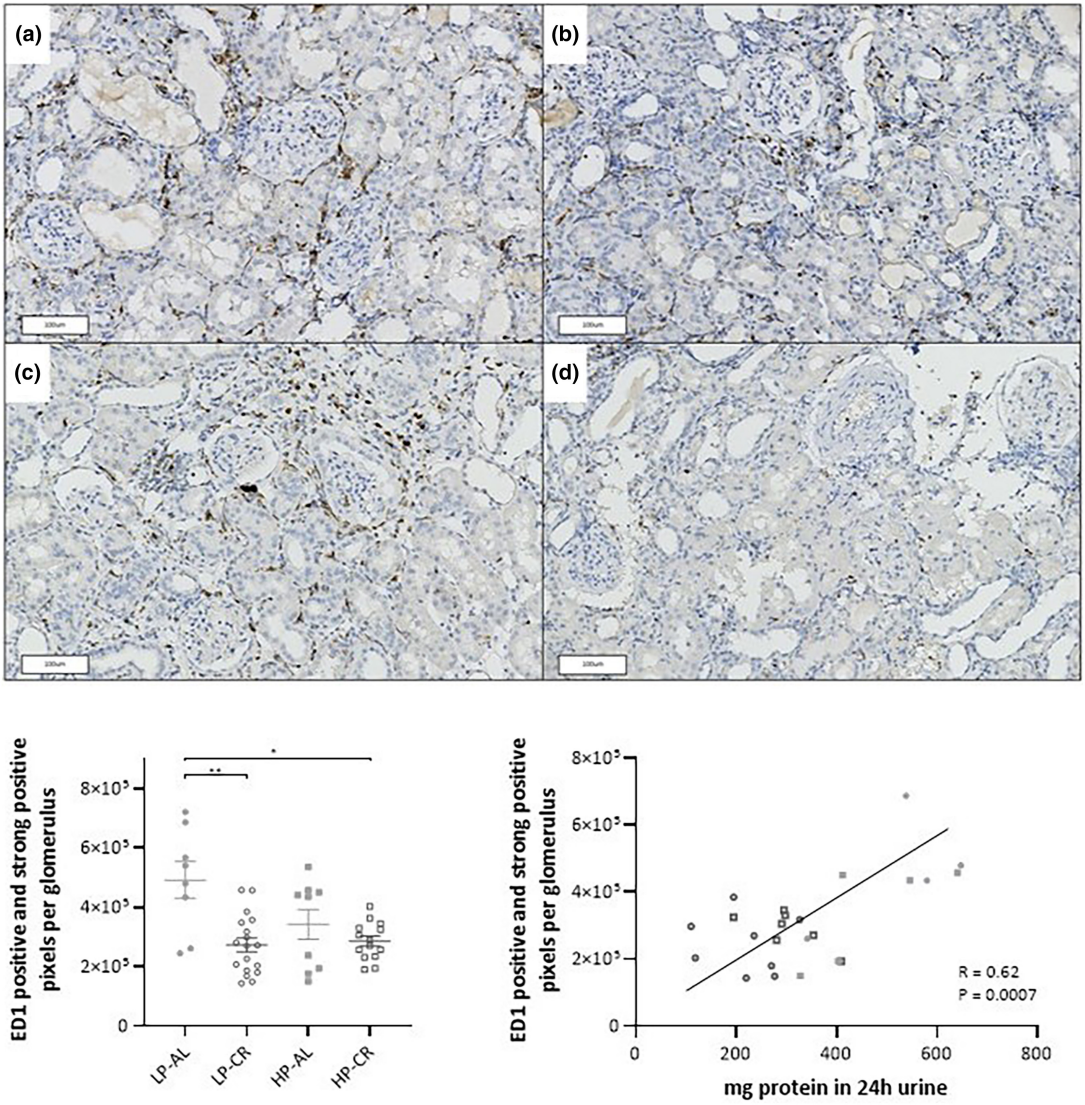
Representative renal sections with ED1 positive cells (brown spots) (a) LP‐AL, (b) LP‐CR, (c) HP‐AL, (d) HP‐CR. (e) Quantitative analysis of ED1 positive cells per glomerulus per group expressed as positive and strong positive pixels per glomerulus. (f) Correlation between ED1 positive cells and proteinuria.● LP‐AL, 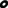 LP‐CR, ■ HP‐AL, 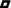 HP‐CR. Data are presented as mean ± SEM. * *p* < 0.05, ** *p* < 0.005.

There were no significant differences in fibrosis or focal glomerulosclerosis observed between the four groups (as shown in Figures S1 and S2). All groups showed a large number of ED1‐positive cells (as shown in Figure 5a–d). The abundance of these cells was so high that manual counting was not feasible. Therefore, the intensity of staining was used to determine the number of positive and strongly positive pixels per glomerulus and per surface as the outcome parameter for the number of glomerular and periglomerular macrophages (Figure 5e) The LP‐CR and the HP‐CR groups exhibited significantly fewer pixels per glomerulus compared to the LP‐AL group. No significant effect was observed between the HP‐AL and the CR groups. A moderately positive correlation (Figure 6f) (*r* = 0.62, *p* < 0.005) was found between glomerular macrophages and proteinuria.

There were no observed differences in the numbers of periglomerular macrophages, focal glomerulosclerosis, or interstitial fibrosis. However, approximately 35% of the kidneys from the AL groups showed signs of tubular dilatation, whereas this was observed in only 13% of the kidneys in the CR groups.

## 4 DISCUSSION

In this study, we observed that CR diminishes proteinuria and lowers blood pressure in rats with established renal disease, even after adjusting for reduced protein intake. Furthermore, the CR groups exhibited smaller kidney volumes and demonstrated lower levels of periglomerular macrophages.

Based on the data, it can be argued that the beneficial effects of CR treatment are likely attributed to a combination of CR itself, protein restriction, and sodium restriction. Remuzzi et al. demonstrated that protein restriction reduces proteinuria and mitigates the severity of kidney disease in Adriamycin‐induced nephropathy (Remuzzi et al., 1985). Additionally, sodium restriction can alleviate renal hypertension, thereby preventing fibrosis of the renal parenchyma (Gomes et al., 2022).

As expected, the treatment's effects are more pronounced in the LP‐CR group, characterized by the lowest intake of protein and sodium. To account for the effects of reduced protein and sodium intake, two additional groups (HP‐AL and HP‐CR) with increased levels of protein and sodium were included in this study. When comparing groups with similar sodium and protein intake (LP‐AL vs. HP‐CR), significantly lower levels of proteinuria and blood pressure were observed in the HP‐CR group, suggesting that CR is the likely cause of reduced proteinuria (Macconi et al., 1997; Poplawski et al., 2011). This similarity in sodium between the LP‐AL and HP‐CR groups was confirmed by sodium excretion, which showed no differences.

The proteinuria‐reducing effect of CR has been demonstrated in several previous studies (Chen et al., 2008; Davis et al., 1983; Gumprecht et al., 1993; Keenan et al., 2000; Klooster, 2013; McKiernan et al., 2007; Wiggins et al., 2005; Xu et al., 2015). Most of these studies induced CR at a young age before the onset of renal dysfunction (Keenan et al., 2000; Wiggins et al., 2005). In a prior investigation, we applied CR to Munich‐Wistar‐Fromter rats with established renal proteinuria, resulting in lower levels of protein excretion compared to control rats, and even to baseline measurements (Klooster, 2013).

However, it is essential to note that the levels of urinary protein excretion in the two studies differed. This discrepancy can be attributed to the use of different animal models (25‐week‐old Munich‐Wistar‐Fromter rats vs. 18‐week‐old Adriamycin‐treated Wistar rats) and variations in the duration of dietary treatment (22 vs. 12 weeks). Munich‐Wistar‐Fromter rats develop proteinuria spontaneously over time (Remuzzi et al., 1992), while Adriamycin‐treated Wistar rats experience renal injury due to the nephrotoxic nature of Adriamycin (Sakemi et al., 1996). This results in a more acute, severe, and adjustable development of proteinuria in the Adriamycin‐induced nephropathy model (Okuda et al., 1986).

A noteworthy detail is the decrease in protein excretion observed between baseline and Week 9. Typically, studies demonstrate a gradual increase in protein excretion following Adriamycin injection. However, this decline in protein excretion has been reported previously by Wang et al. but is not explained (Wang et al., 2000).

Blood pressure was significantly lower in CR groups compared to AL groups. However, it's essential to note that overall blood pressure was lower than expected, potentially due to the impact of anesthesia on the cardiovascular system (Zutphen et al., 2001). Given that all groups were exposed to isoflurane similarly, it is likely that the observed differences between groups are treatment effects. To mitigate the influence of isoflurane in future studies, we recommend employing telemetry for blood pressure recording (Braga & Prabhakar, 2009) or using the tail cuff method (Erken et al., 2013).

Several studies indicate that CR can normalize blood pressure (Braga & Prabhakar, 2009; De Souza Nunes Faria et al., 2022; Di Daniele et al., 2021; Erken et al., 2013; Gomes et al., 2022; Juárez‐Orozco et al., 2015; Klooster, 2013; Macconi et al., 1997; Melenhorst et al., 2006; Nicoll & Henein, 2018; Okuda et al., 1986; Poosti et al., 2012; Poplawski et al., 2011; Remuzzi et al., 1985, 1992; Sakemi et al., 1996; Snijder et al., 2014; van Goor et al., 1991; Wang et al., 2000; Zutphen et al., 2001), and the reduction in blood pressure (hypotension) may slow down the progression of kidney disease. In our study, a clear correlation between proteinuria and blood pressure are observed. From this information, it can be argued that CR affects blood pressure, and not necessarily through a direct impact on urinary protein excretion. Remuzzi et al. demonstrated that blood pressure reduction alone was not responsible for the improvement in protein filtration at the glomerular level (Remuzzi et al., 1999). Moreover, blood pressure reduction achieved with a calcium channel blocker did not induce any decrease in proteinuria (Remuzzi et al., 1994). Unfortunately, our study did not include an additional group to rule out low blood pressure as a contributing factor.

In addition to CR, blood pressure reduction could potentially be influenced by reduced sodium intake or reduced fluid intake due to CR treatment. Sodium excretion was similar between the LP‐AL and HP‐CR groups, and urine production did not change over time in the CR groups, suggesting that the impact on blood pressure was minor.

Decreased creatinine clearance, kidney perfusion, and increased plasma creatinine levels suggested an overall decline in kidney function despite the CR treatment. The lack of significant differences in fibrosis and focal glomerulosclerosis between the AL and CR groups, except for smaller kidney volume, indicates that the reduction in proteinuria was not primarily due to structural and functional changes in the kidneys.

Usually, creatinine clearance is corrected for kidney weight. However, due to the need for repeated measurements, it was not possible to collect kidney weight repetitively over time. Therefore, the PET scan was used to calculate kidney volume. These calculations may be biased due to partial volume (Hoffman et al., 1979; Kessler et al., 1984) and spillover effects. Unfortunately, we did not measure kidney weight at the end of the study and because of that we were not able to correlate kidney weight with the volume estimation from the PET scan. Therefore, we recommend using computed tomography or magnetic resonance imaging in future studies to determine kidney volume more precisely (Almajdub et al., 2008).

The image analysis differed from the method described by Juárez‐Orozco et al. (2015). In the present study, only the initial 2 min of the total acquisition time were used for analysis to avoid potential effects of radioactive metabolites (Müller et al., 1994; Rosenspire et al., 1990). As only the first 2 min were used, there was insufficient data to fit for two compartments, and consequently, a one‐tissue compartment model was used. Additionally, the lumen of the abdominal aorta was used for the image‐derived input function instead of the left ventricle, as the heart was not in the field of view of the scanner. The lumen of the aorta is much smaller than the lumen of the left ventricle and is therefore more vulnerable to noise.

ED‐1 staining showed significantly higher numbers of periglomerular macrophages, mainly in the LP‐AL group. Infiltration of macrophages is often observed in CKD. Lan et al. demonstrated a correlation between macrophage infiltration and kidney dysfunction, such as proteinuria (Lan et al., 1991). Shimada et al. stated that an increase in renal perfusion pressure causes activation of mechanistic targets of rapamycin complex 1, resulting in the infiltration of macrophages and monocytes into the kidney (Shimada et al., 2022).

Except for the ED‐1 staining, no differences were found between groups in both PAS and α‐SMA staining. This finding was unexpected and not in line with our previous study where we observed lower focal glomerulosclerosis and fibrosis in the CR groups compared to the AL groups (Klooster, 2013). Gumprecht et al. also observed minimal histologic changes characteristic of CKD in dietary‐restricted rats (Gumprecht et al., 1993). The lack of positive staining in the present study may be attributed to the study design, as immunohistochemistry was only performed on kidneys from rats that survived until the end of the study.

## 5 LIMITATIONS

A limitation of the study was the low number of animals that reached the study endpoint. Due to side effects from Adriamycin‐induced nephropathy, over half of the animals needed to be euthanized. Despite using a relatively low dose of Adriamycin, the levels of proteinuria were higher than anticipated, as noted in a pilot study aimed at determining the appropriate Adriamycin dose.

While the conclusions drawn from the study are clear, a larger number of animals per group could have provided a more comprehensive understanding of the outcomes from the immunohistochemistry data. Additionally, including an extra group to assess blood pressure as an independent variable would contribute to a better understanding of the impact of CR on urinary protein excretion.

## 6 CONCLUSION

CR has been shown to decrease proteinuria and lower blood pressure in rats with established renal disease, even when considering reduced protein and sodium intake. Further experiments are required to thoroughly investigate and comprehend the influence of CR on blood pressure in conjunction with protein excretion and glomerular macrophages.

This preclinical study can serve as a foundational basis for future human studies exploring the potentially beneficial effects of CR in renal disease. It may ultimately guide intervention studies in patients with proteinuric and obesity‐related nephropathy.

## AUTHOR CONTRIBUTIONS

J.W.A.S., A.K., H.v.G., and S.J.L.B. were responsible for the study concept, design, and methodology. J.W.A.S., A.v.W., I.K., and A.K. were responsible for the data acquisition, processing, analysis, interpretation, and statistical analysis. A.L., B.L.G., and J.W.A.S. were responsible for processing, analysis, and interpretation of the PET data. R.S., R.D., H.B., A.v.W., A.K., H.v.G., and S.J.L.B. supervised the study. All authors contributed to the article.

## FUNDING INFORMATION

This research received no external funding.

## CONFLICT OF INTEREST STATEMENT

The authors declare no conflict of interest.

## ETHICS STATEMENT

The experimental protocol was according to the ARRIVE guidelines and approved by the CentralCommittee on Animal Experiments of The Netherlands (license number AVD105002016663) and the animal welfare body of the University Medical CenterGroningen (protocol 16663‐01‐001) according to directive 2010/63/EU of theEuropean Parliament.

## Supporting information


Data S1:

## Data Availability

Data reported in this paper are archived in the University Medical Center Groningen, University of Groningen and are available on request.
