# Supplementary material for: Caloric restriction reduces proteinuria in male rats with established nephropathy
Source: Physiol Rep. 2024 Mar 5;12(5):e15942. doi: 10.14814/phy2.15942 (PMC10912948; doi:10.14814/phy2.15942)
Supplement: Supplementary file 1 — Data S1: [file PHY2-12-e15942-s001.docx]

**Supplementary data**

**Caloric restriction reduces proteinuria in rats with established nephropathy**

**J.W.A. Sijbesma**

***Supplementary table S1.*** *Composition of the 4 diets used in this study. All diets were obtained from Special Diets Services (SDS), Witham, United Kingdom*

| **Diet** | **RB 12%** | **RB 12% +Vits/mins** | **RB 20%** | **RB 20% +Vits/mins** |
| --- | --- | --- | --- | --- |
| **Diet code** | **824245** | **824252** | **824250** | **824254** |
| **Gross Energy Kcal/kg** | 3914 | 3834 | 3886 | 3806 |
| **Ingredients (%)** |  |  |  |  |
| **Moisture** | 3.89 | 3.80 | 4.51 | 4.41 |
| **Crude oil** | 7.31 | 7.30 | 7.43 | 7.42 |
| **Crude protein** | 12.02 | 12.01 | 20.00 | 19.99 |
| **Crude fibre** | 3.50 | 3.50 | 3.50 | 3.50 |
| **Ash** | 3.19 | 5.04 | 3.42 | 5.26 |
| **NFE** | 69.4 | 67.4 | 60.5 | 58.5 |
| **Pectine** | 0.00 | 0.00 | 0.00 | 0.00 |
| **Hemicellulose** | 0.10 | 0.10 | 0.10 | 0.10 |
| **Cellulose** | 4.80 | 4.80 | 4.80 | 4.80 |
| **Lignin** | 0.00 | 0.00 | 0.00 | 0.00 |
| **Starch** | 42.6 | 40.2 | 35.4 | 33.0 |
| **Sugar** | 25.3 | 25.8 | 23.5 | 23.9 |

***Supplementary table S2.*** *Number of animals (N) included in the measurements per group per time point.*

|  | **Timepoints** | | |
| --- | --- | --- | --- |
| **Groups** | **Baseline** | **9 weeks** | **12 weeks** |
| **LP-AL** | 14 | 8 | 5 |
| **LP-CR** | 14 | 10 | 9 |
| **HP-AL** | 12 | 7 | 4 |
| **HP-CR** | 14 | 8 | 6 |

**
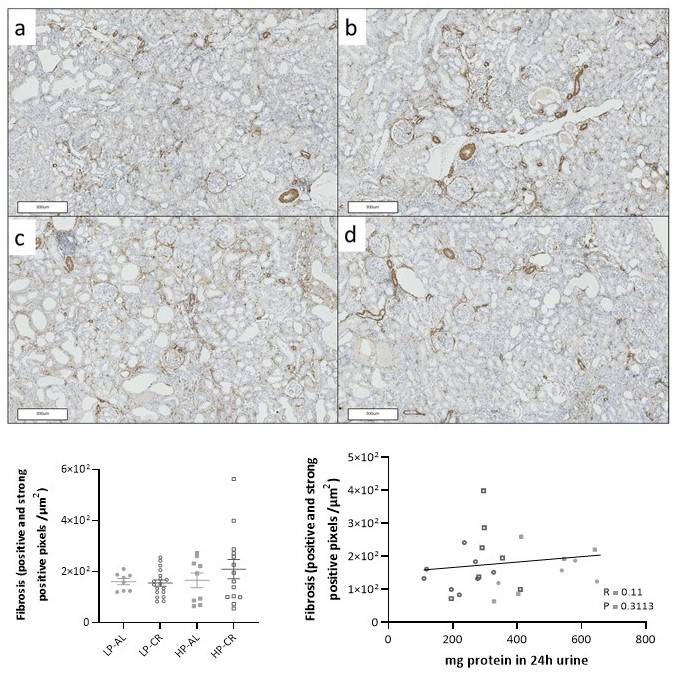
**

***Supplementary figure S1.*** *Representative renal sections with fibrosis (α-SMA positive sections, brown spots) (****a****, LP-AL,* ***b****, LP-CR,* ***c****, HP-AL,* ***d****, HP-CR). (****e****) Quantitative analysis of α-SMA positive sections per group expressed as positive and strong positive pixels per µm^2^.* *(****f****) Correlation between fibrosis and proteinuria ● LP-AL, 🞇 LP-CR, ■ HP-AL, 🞑 HP-CR. Data is presented as mean ± SEM. * p < 0.05, ** p < 0.005.*


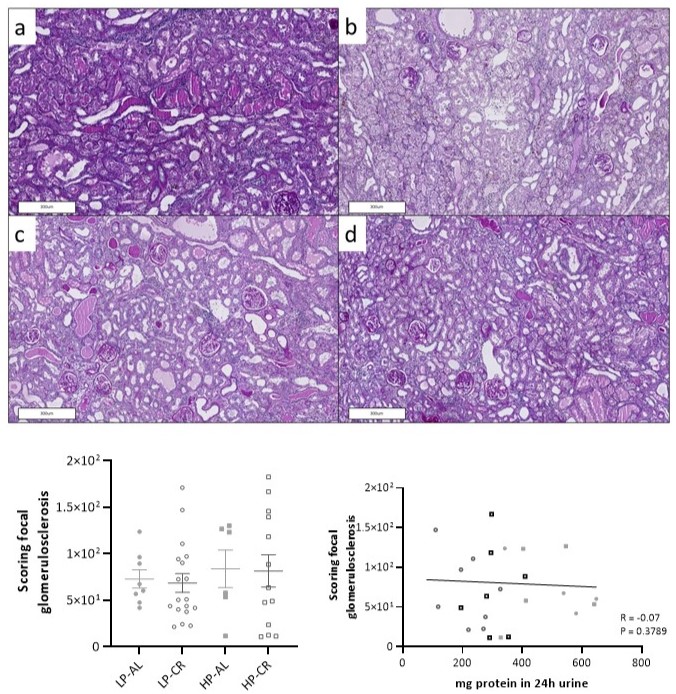


***Supplementary figure S2.*** *Representative renal sections with PAS staining (****a****, LP-AL,* ***b****, LP_CR,* ***c****, HP-AL,* ***d****, HP-CR). (****e****) Quantitative analysis of focal glomeruosclerosis scored as described by van Goor et al. ^26^. (****f****) Correlation between focal glomeruosclerosis and proteinuria ● LP-AL, 🞇 LP-CR, ■ HP-AL, 🞑 HP-CR. Data is presented as mean ± SEM. * p < 0.05, ** p < 0.005.*
